# Supplementary material for: Synthesis, Biological Evaluation, DNA Binding, and Molecular Docking of Hybrid 4,6-Dihydrazone Pyrimidine Derivatives as Antitumor Agents
Source: Molecules. 2022 Dec 26;28(1):187. doi: 10.3390/molecules28010187 (PMC9822369; doi:10.3390/molecules28010187)
Supplement: Supplementary file 1 [file molecules-28-00187-s001.zip › molecules-2086281-supplementary.pdf]

Supplementary materials

Synthesis, Biological Evaluation, DNA Binding, and Molecular Docking of Hybrid 4,6-Dihydrazone Pyrimidine Derivatives as Antitumor Agents

Hairong Lan [1], Junying Song [2], Juan Yuan [1,*], Aiping Xing [1], Dai Zeng [1], Yating Hao [1], Zhenqiang Zhang [2,*] and Shuying Feng [3]

Table of contents

Figure S1. ^{1}H NMR spectrum of **10a**.

Figure S2. ^{13}C spectrum of **10a**

Figure S3. ESI-HRMS spectrum of **10a**.

Figure S4. IR spectrum of **10a**.

Figure S5. ^{1}H NMR spectrum of **10b**.

Figure S6. ^{13}C spectrum of **10b**.

Figure S7. ESI-HRMS spectrum of **10b**.

Figure S8. IR spectrum of **10b**.

Figure S9. ^{1}H NMR spectrum of **10c**.

Figure S10. ^{13}C spectrum of **10c**.

Figure S11. ESI-HRMS spectrum of **10c**.

Figure S12. IR spectrum of **10c**.

Figure S13. ^{1}H NMR spectrum of **10d**.

Figure S14. ^{13}C spectrum of **10d**.

Figure S15. ESI-HRMS spectrum of **10d**.

Figure S16. IR spectrum of **10d**.

Figure S17. ^{1}H NMR spectrum of **10e**.

Figure S18. ^{13}C spectrum of **10e**.

Figure S19. ESI-HRMS spectrum of **10e**.

Figure S20. IR spectrum of **10e**.

Figure S21. ^{1}H NMR spectrum of **10f**.

Figure S22. ^{13}C spectrum of **10f**.

Figure S23. ESI-HRMS spectrum of **10f**.

Figure S24. IR spectrum of **10f**.

Figure S25. UV-vis spectra of **10a**~**10f**.

Figure S26. UV-vis spectra of **10a**.

Figure S27. UV-vis spectra of **10b**.

Figure S28. UV-vis spectra of **10c**.

Figure S29. UV-vis spectra of **10d**.

Figure S30. UV-vis spectra of **10e**.

Figure S31. UV-vis spectra of **10f**.

Figure S32. Visualizations of **10a** docking with DNA (PDB ID: 1D18).

Figure S33. Visualizations of **10f** docking with DNA (PDB ID: 1D18).

Table S1. The ΔG_b^0 and K_b of **10a** and **10f** interacting with DNA (PDB ID: 1D18).

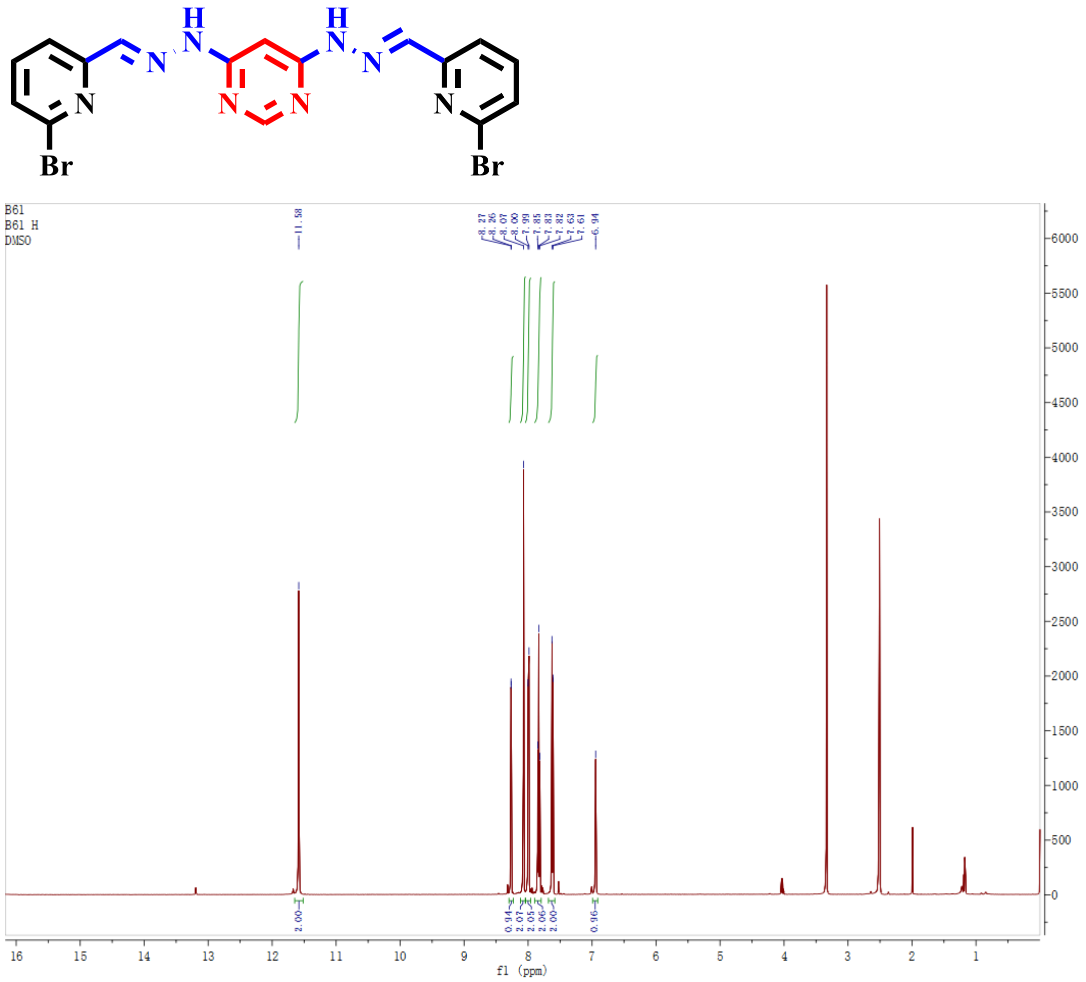

Figure S1. ^{1}H NMR spectrum of **10a** in DMSO-d6.

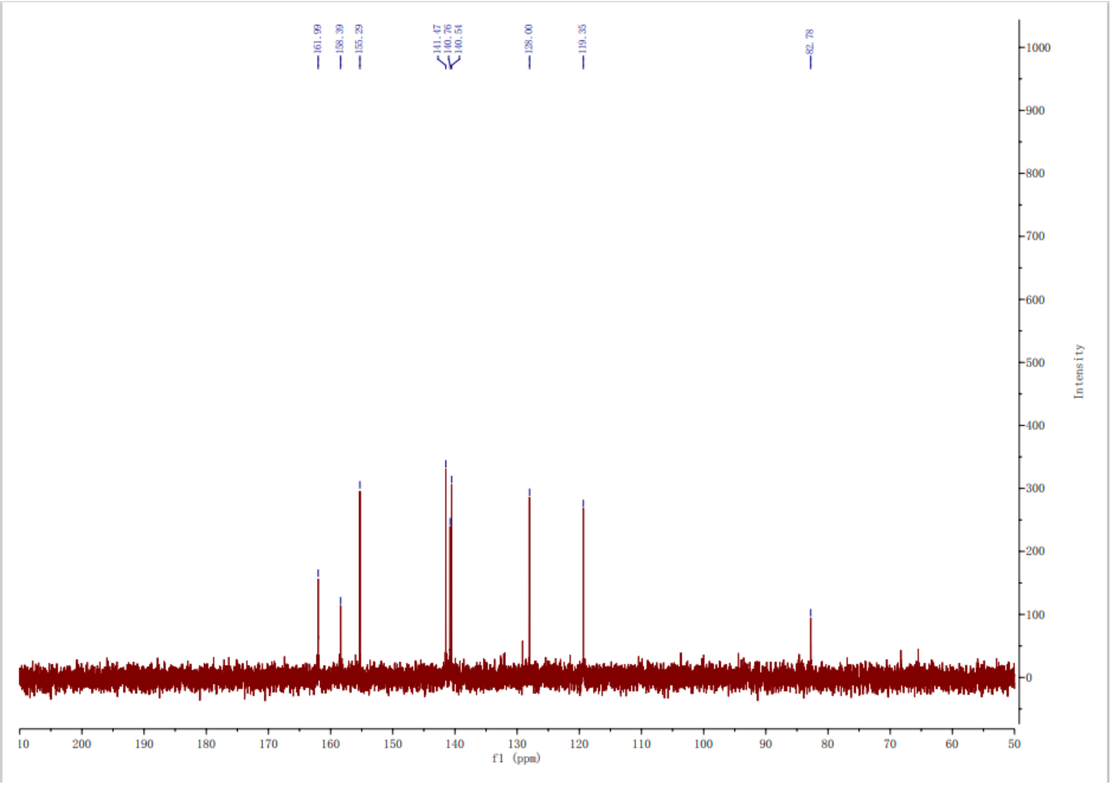

Figure S2. ^{13}C spectrum of **10a** in DMSO-d6.

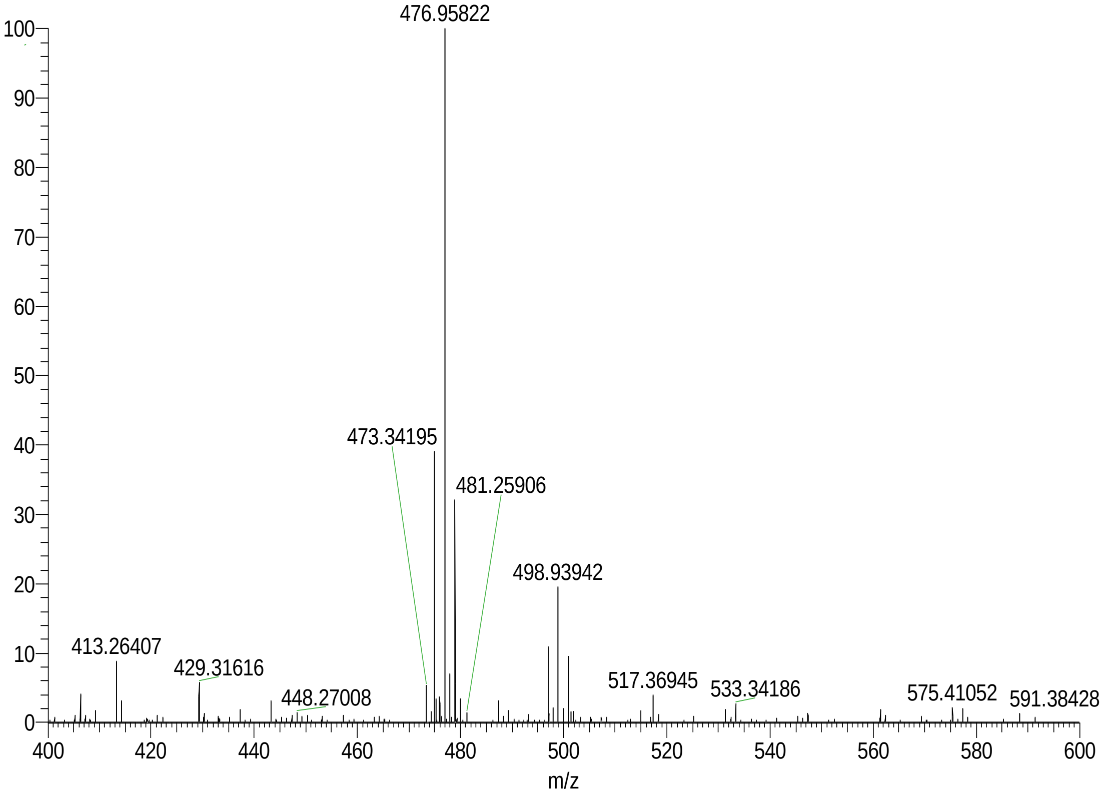

Figure S3. ESI-HRMS of **10a** at m/z 476.95822 for $C_{16}H_{12}Br_2N_8$ $[M+H]^+$.

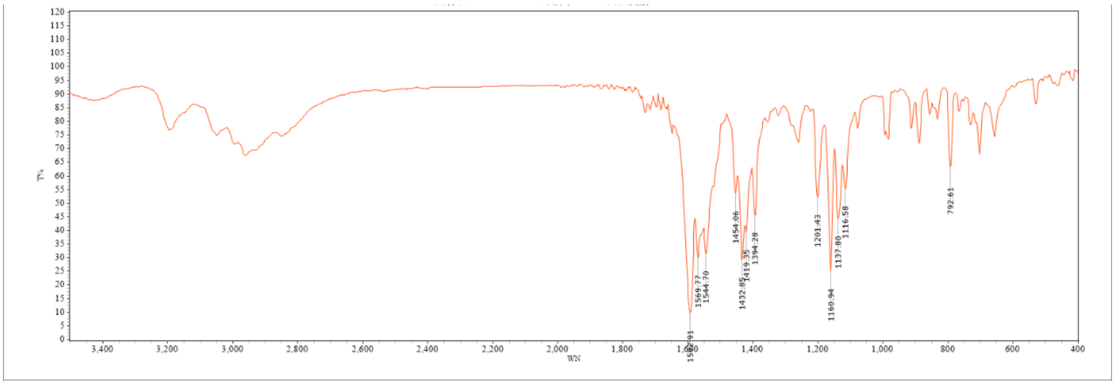

Figure S4. IR spectrum of **10a**.

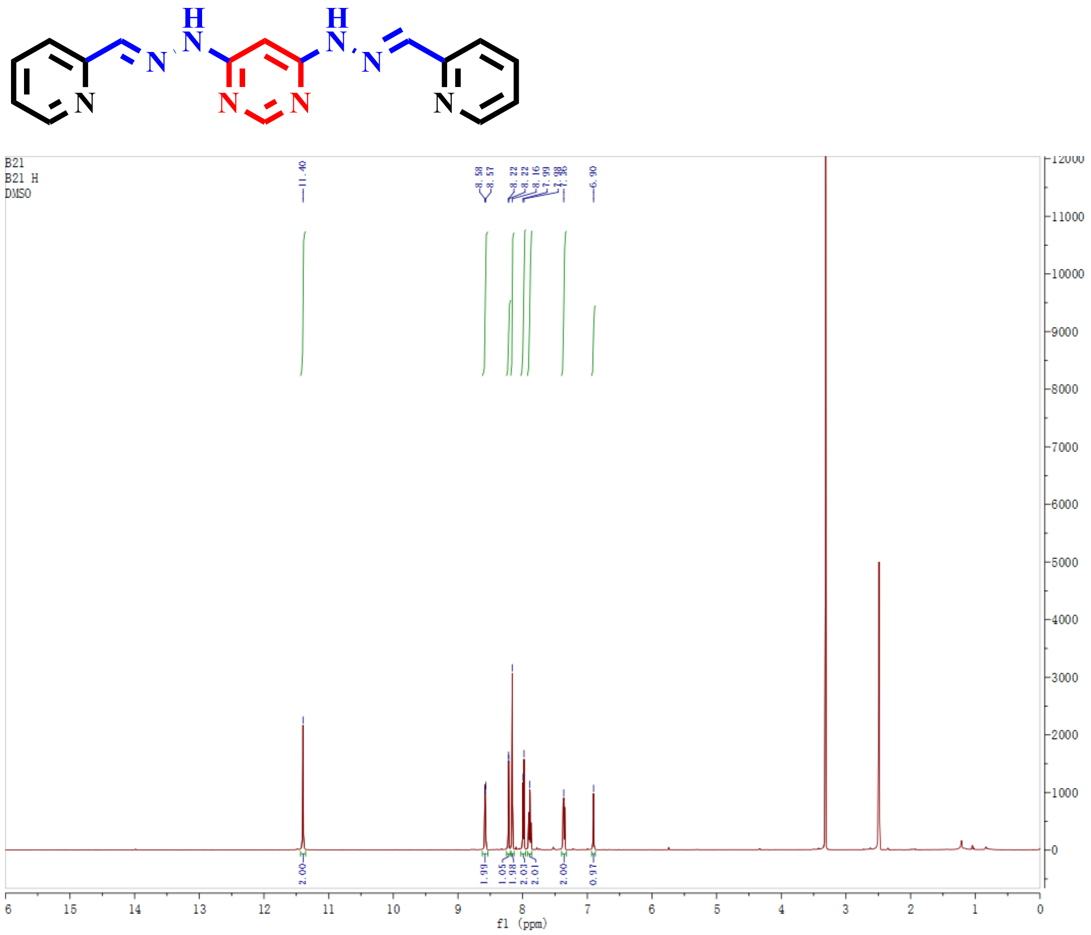

Figure S5. ^{1}H NMR spectrum of **10b** in DMSO-d6.

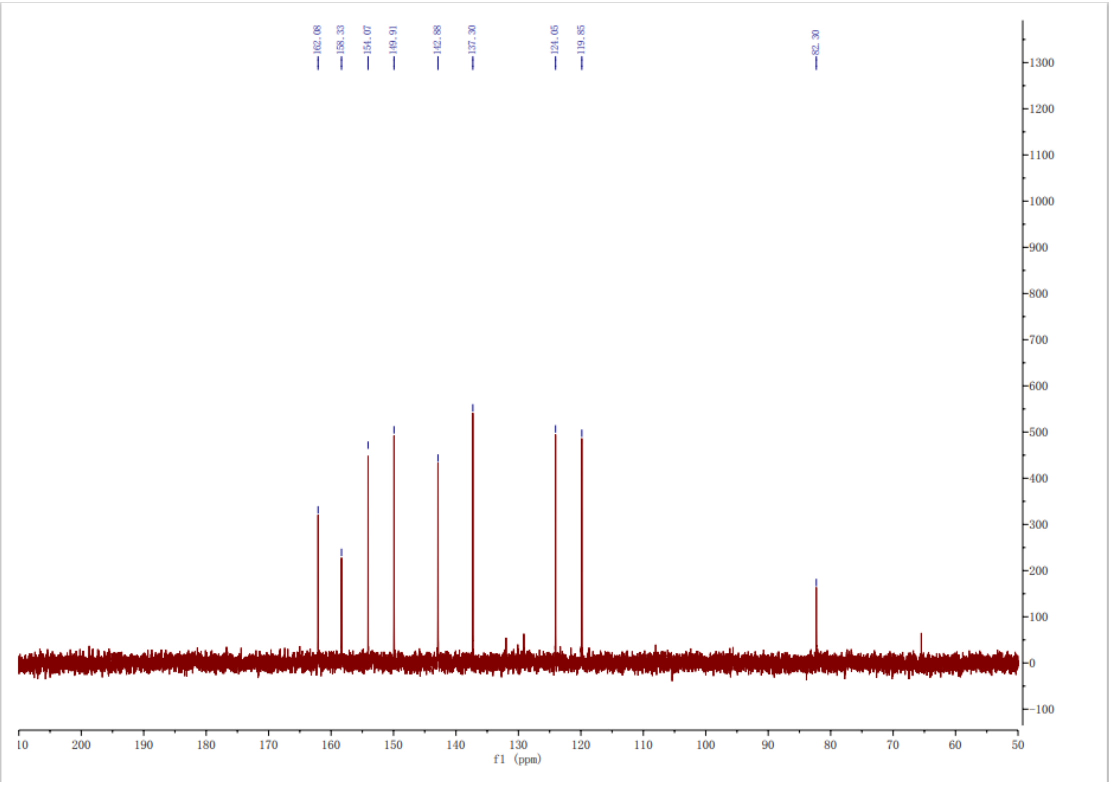

Figure S6. ^{13}C spectrum of **10b** in DMSO-d6.

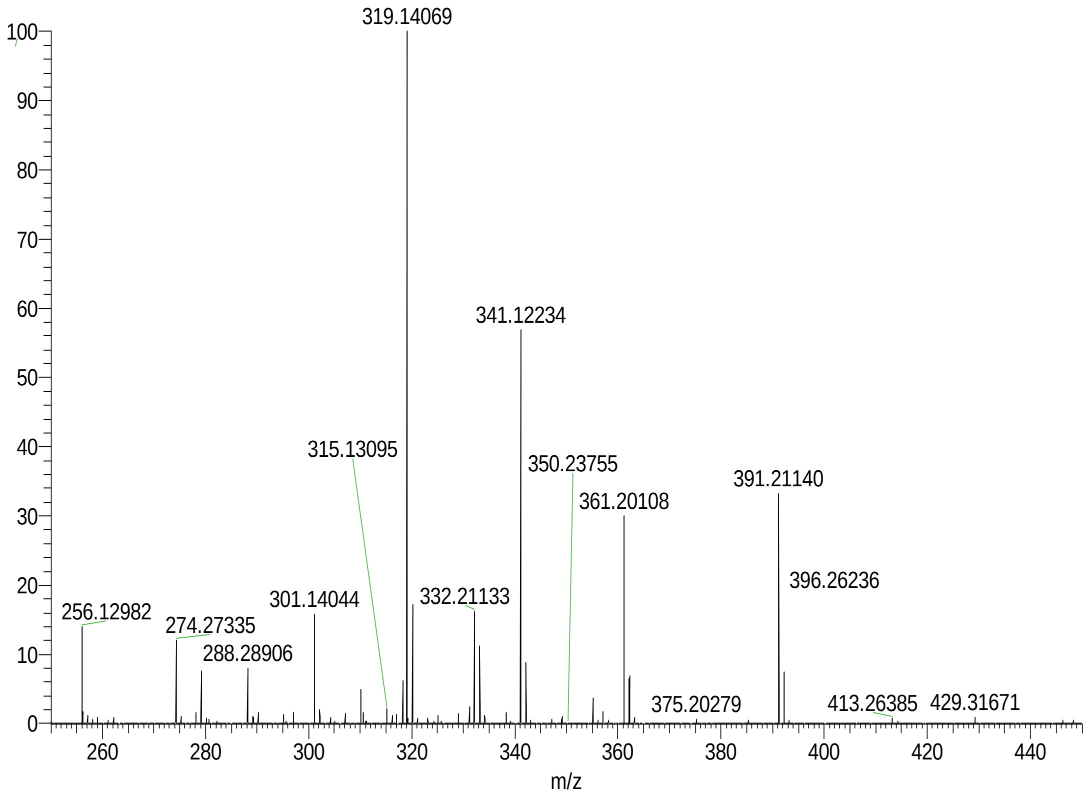

Figure S7. ESI-HRMS of **10b** at m/z 319.14069 for $C_{16}H_{14}N_8$ $[M+H]^+$.

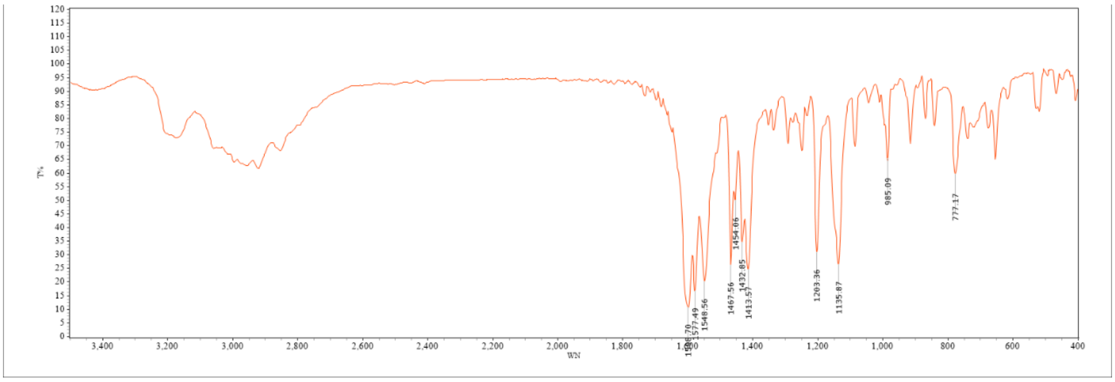

Figure S8. IR spectrum of **10b**.

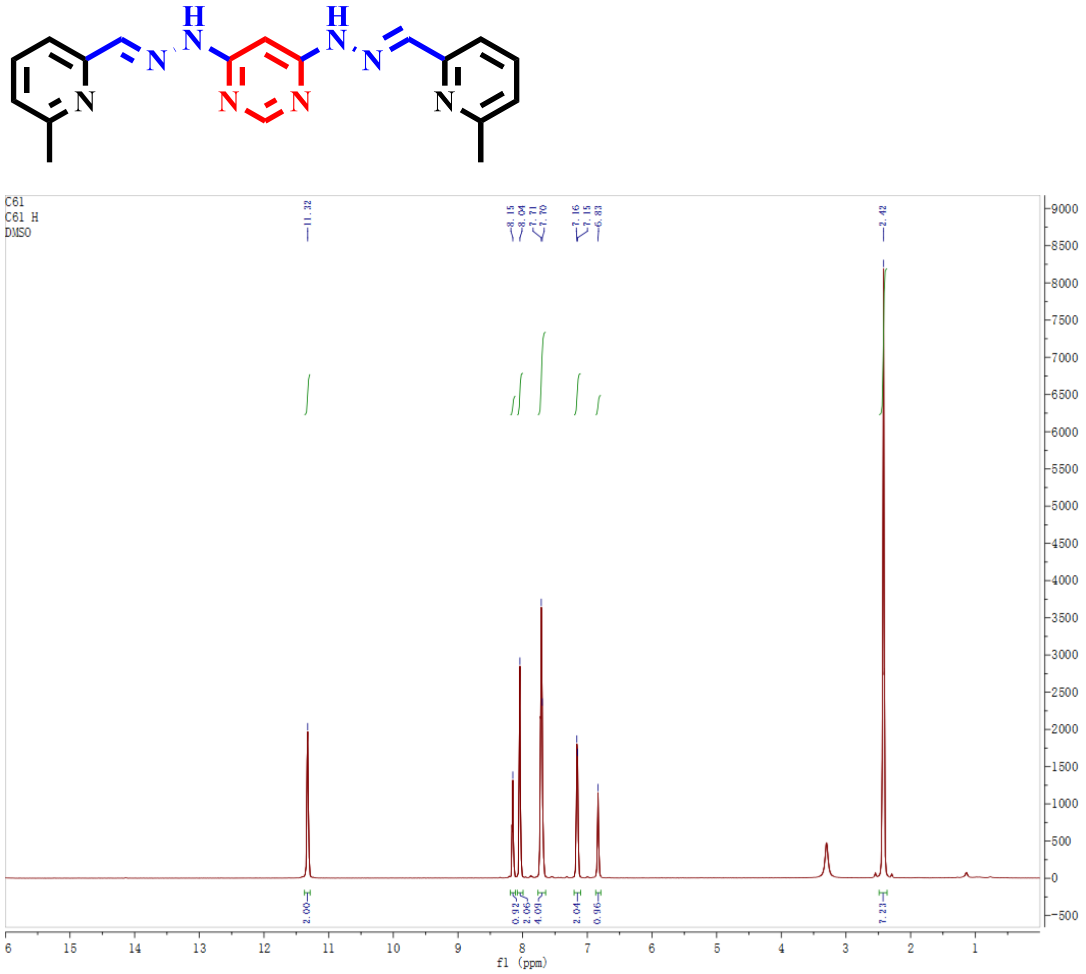

Figure S9. ^{1}H NMR spectrum of **10c** in DMSO-d6.

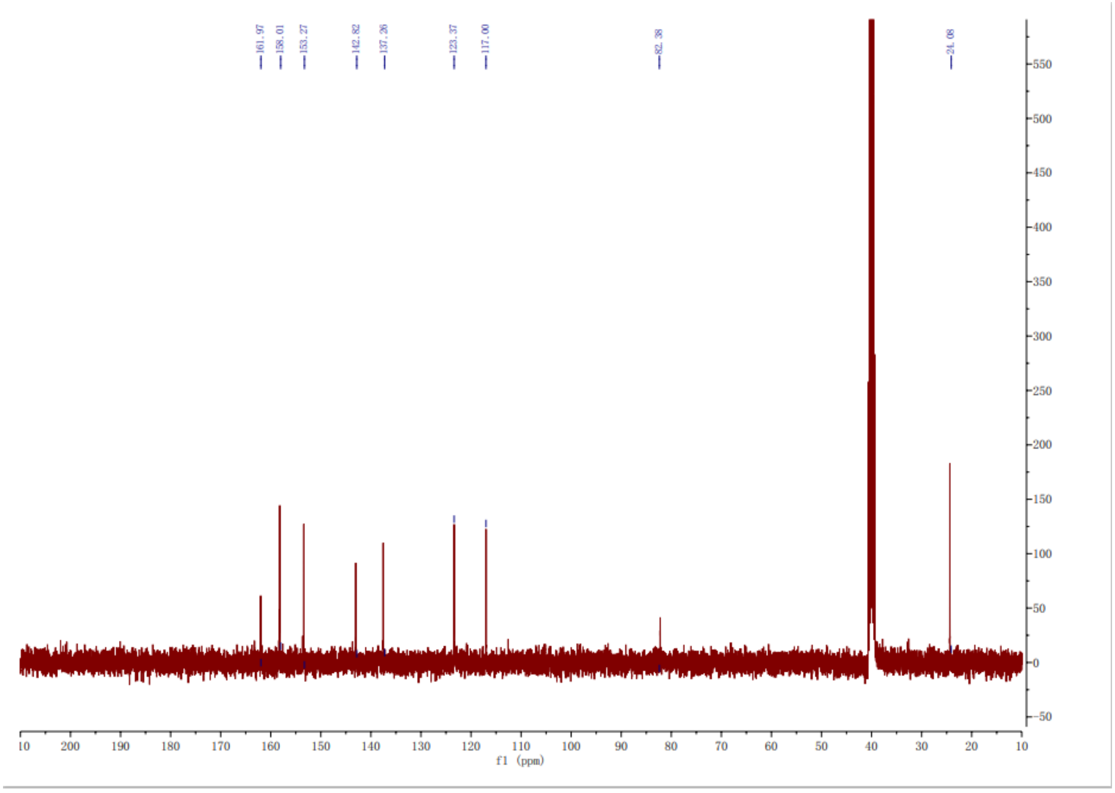

Figure S10. ^{13}C spectrum of **10c** in DMSO-d6.

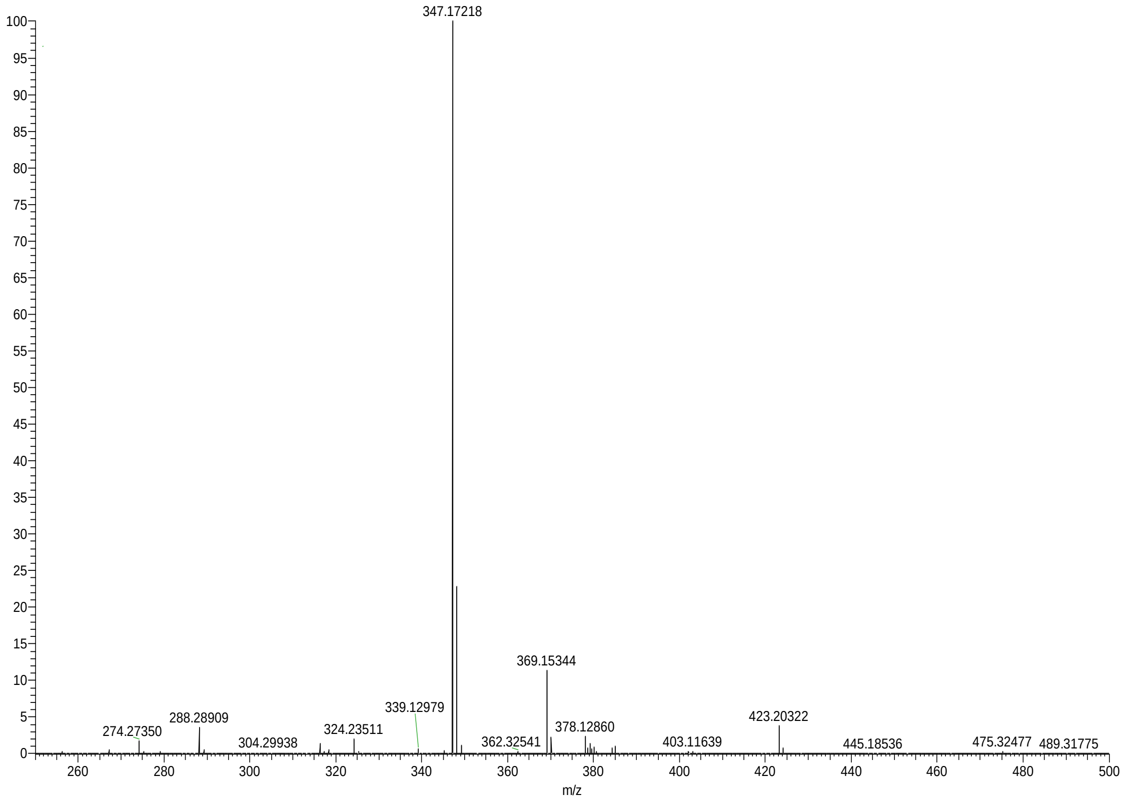

Figure S11. ESI-HRMS of **10c** at m/z 347.17218 for $C_{18}H_{18}N_8$ $[M+H]^+$.

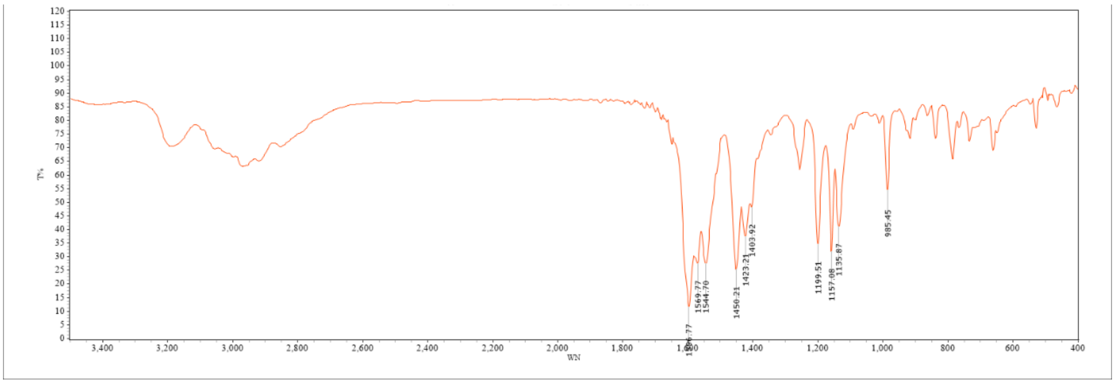

Figure S12. IR spectrum of **10c**.

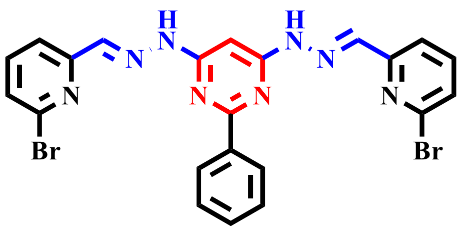

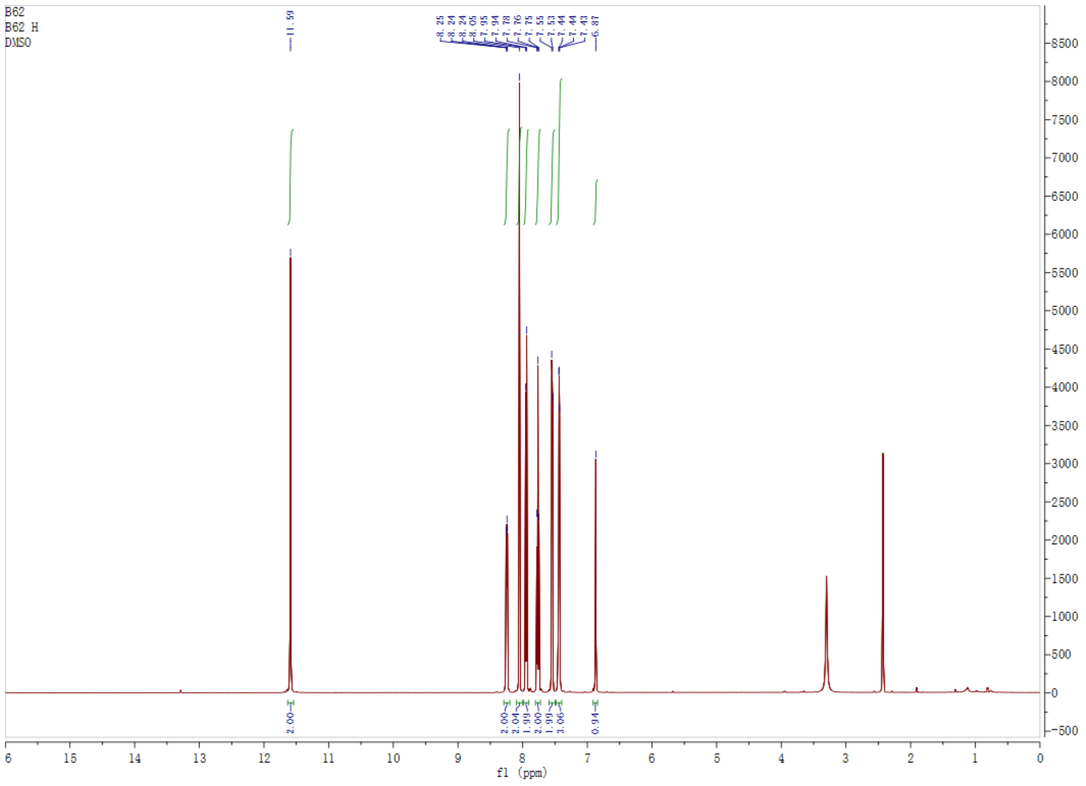

Figure S13. ^{1}H NMR spectrum of **10d** in DMSO-d6.

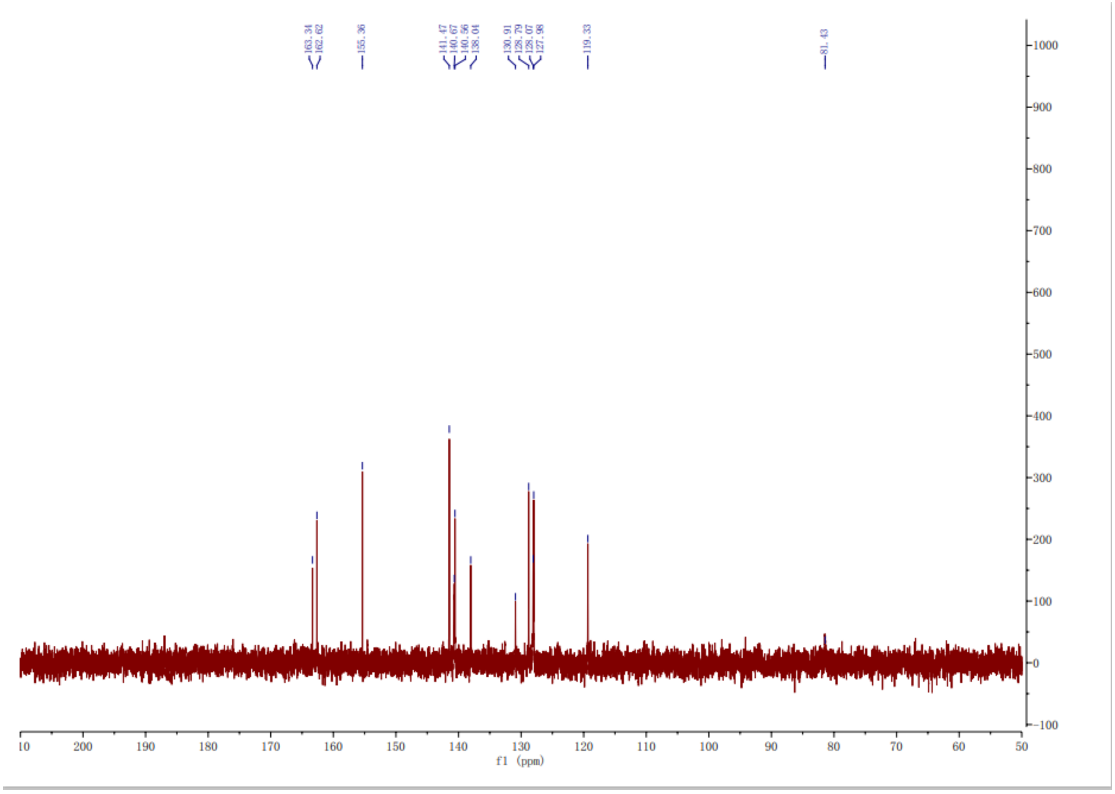

Figure S14. ^{13}C spectrum of **10d** in DMSO-d6.

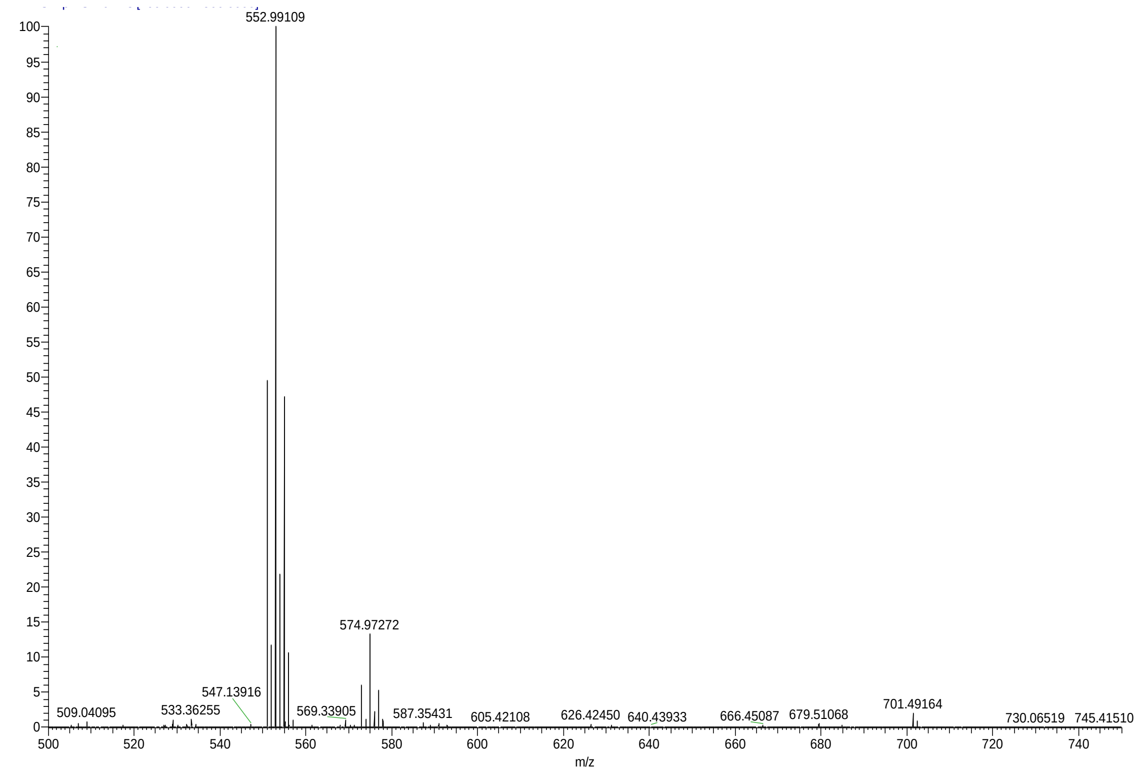

Figure S15. ESI-HRMS of **10d** at m/z 552.99109 for $C_{22}H_{16}Br_2N_8$ $[M+H]^+$.

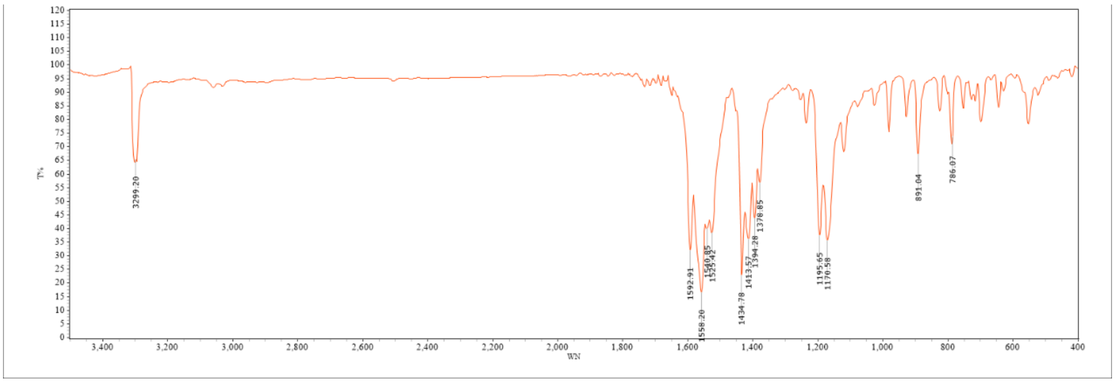

Figure S16. IR spectrum of **10d**.

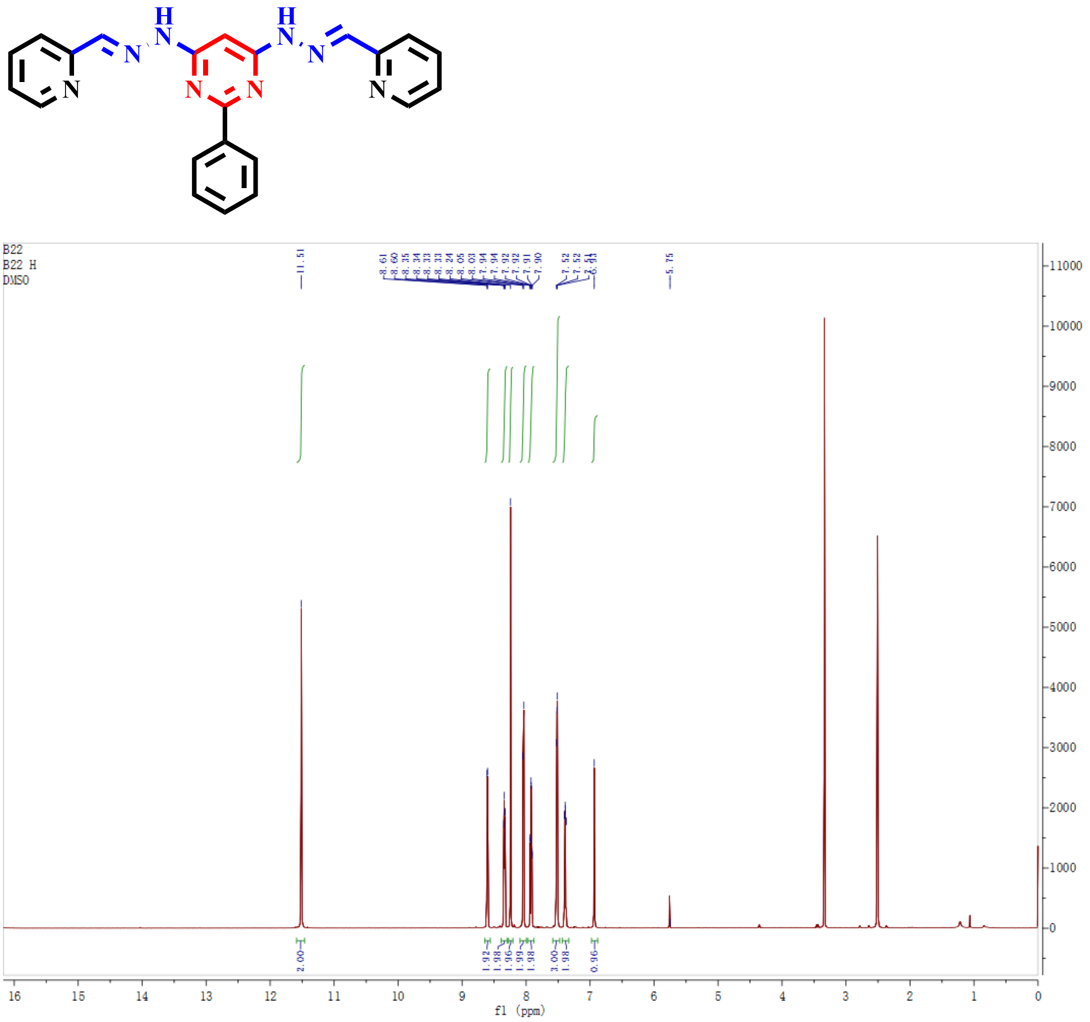

Figure S17. ^{1}H NMR spectrum of **10e** in DMSO-d6.

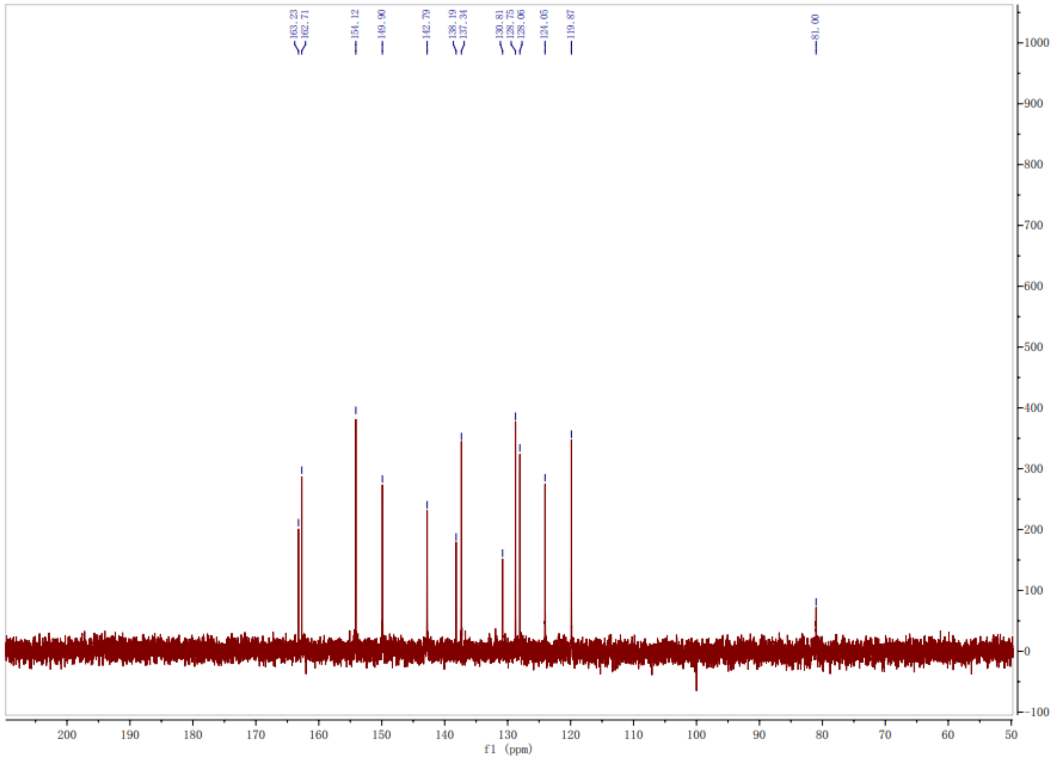

Figure S18. ^{13}C spectrum of **10e** in DMSO-d6.

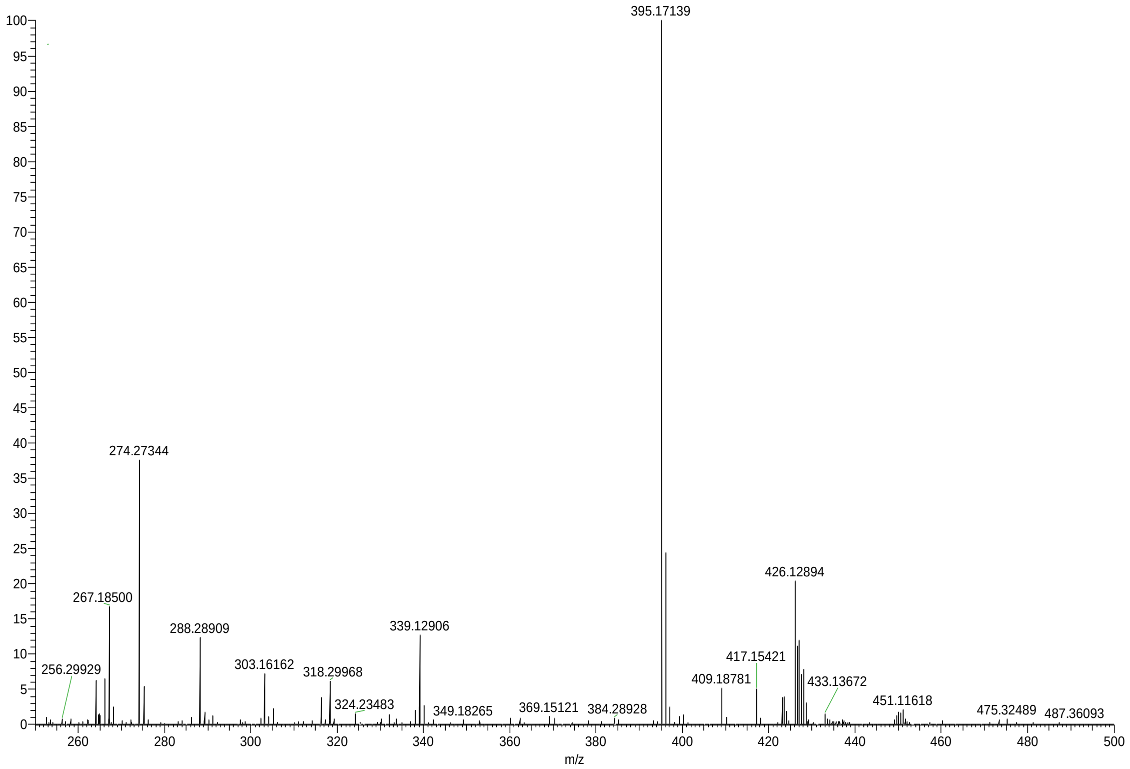

Figure S19. .ESI-HRMS of **10e** at m/z 395.17139 for $C_{22}H_{18}N_8$ [M+H]$^+$.

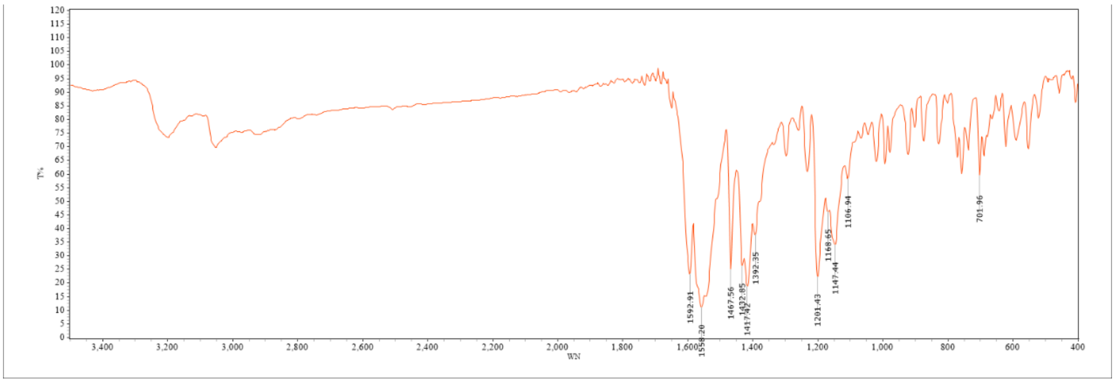

Figure S20. IR spectrum of **10e**.

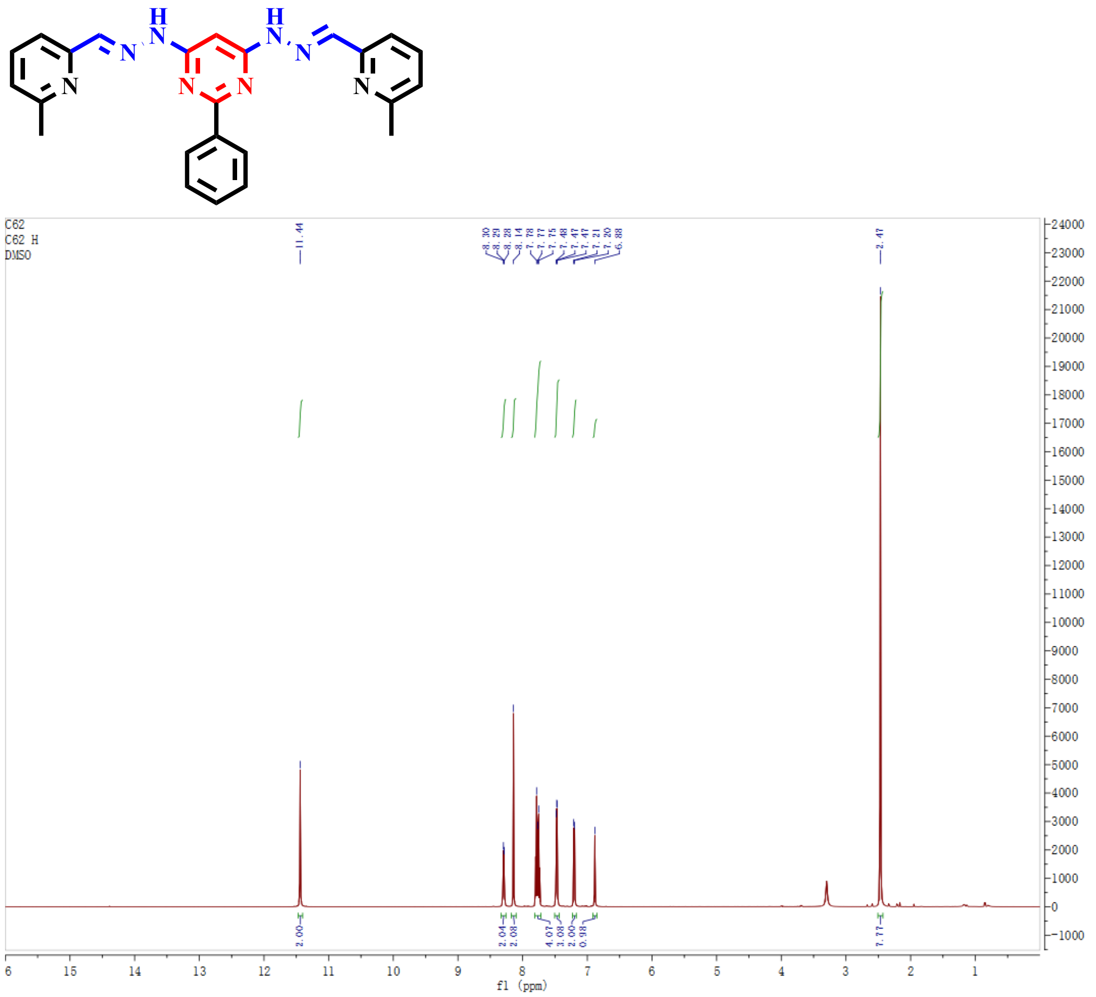

Figure S21. ^1H NMR spectrum of **10f** in DMSO-d6.

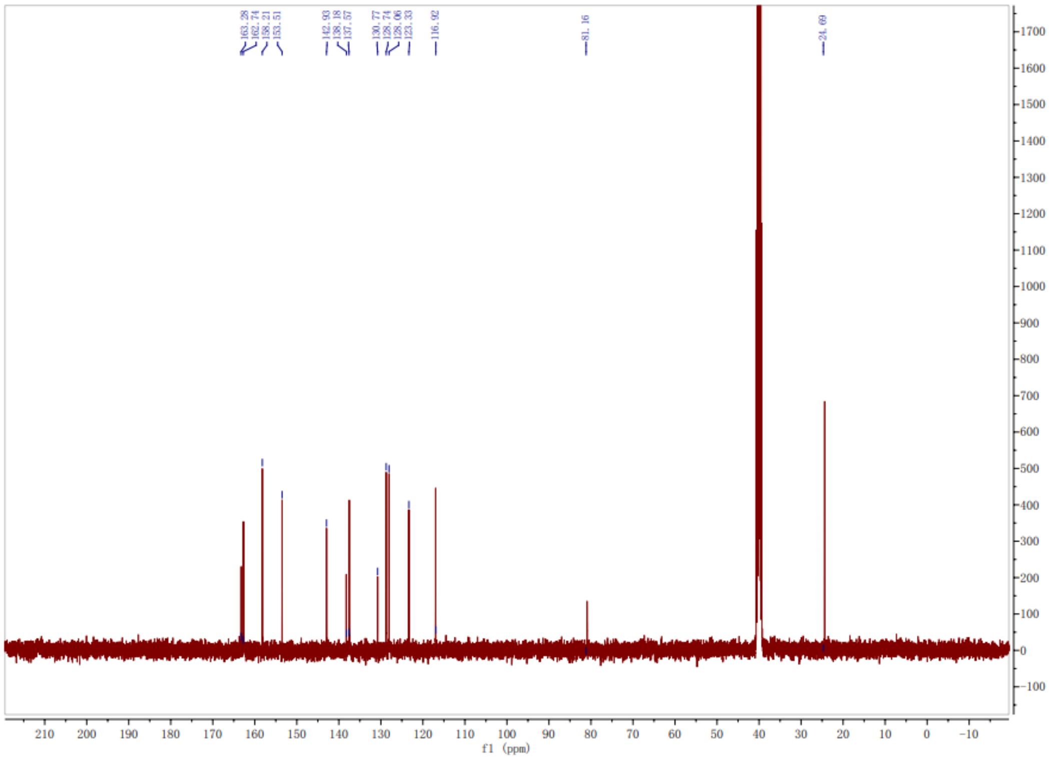

Figure S22. ^{13}C spectrum of **10f** in DMSO-d6.

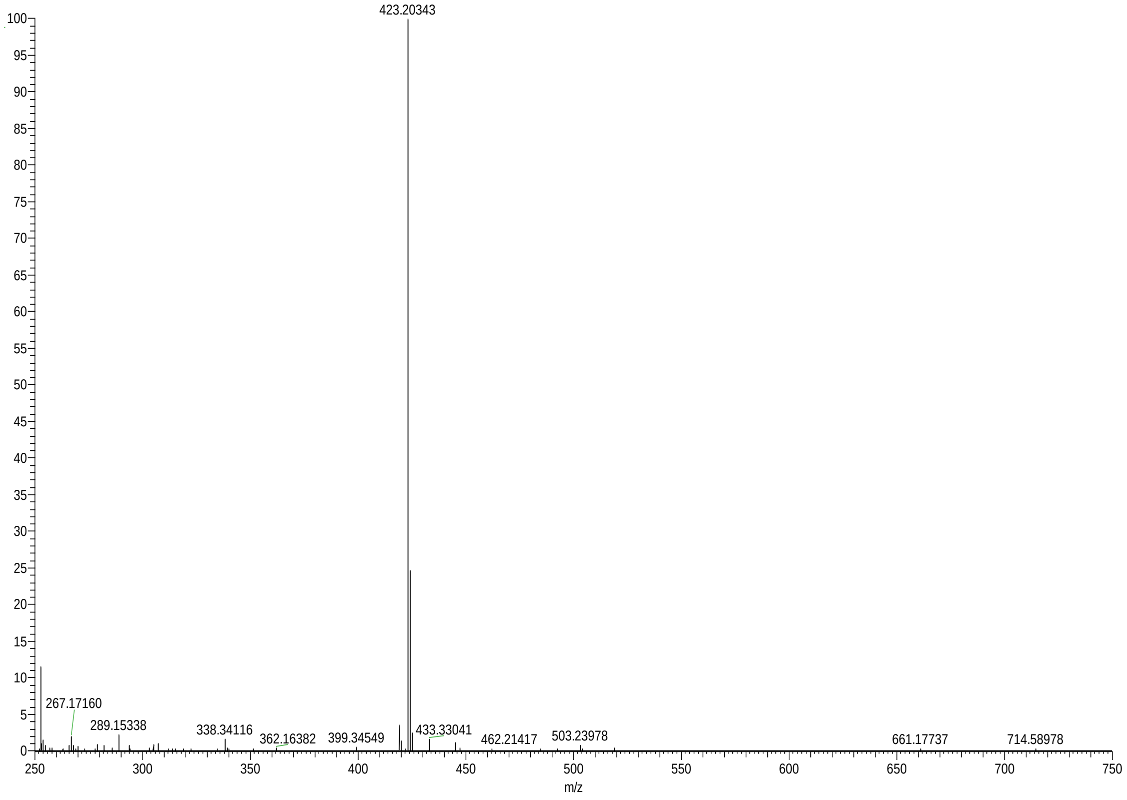

Figure S23. ESI-HRMS of **10f** at m/z 423.20343 for $C_{24}H_{22}N_8$ $[M+H]^+$.

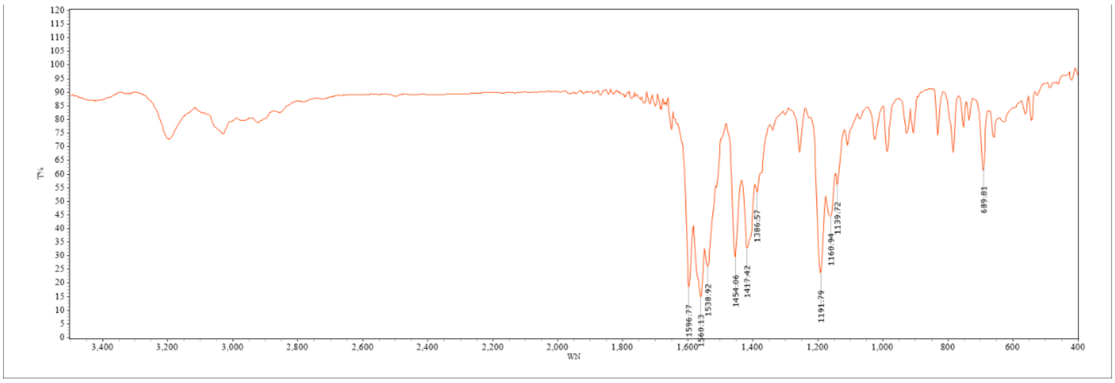

Figure S24. IR spectrum of **10f**.

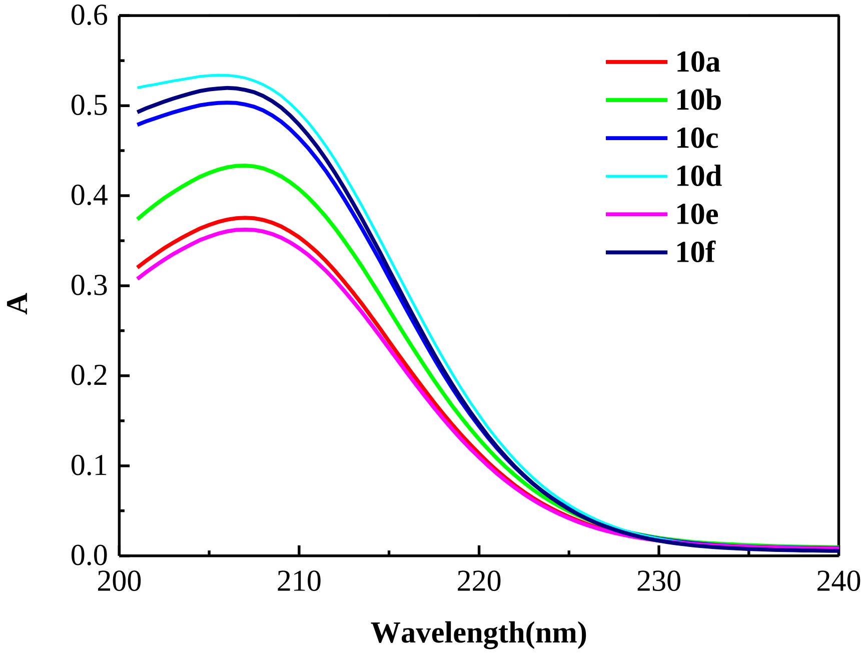

Figure S25. UV-vis spectra of **10a~10f** at 0.1 μM in Tris-HCl buffer (pH 7.4).

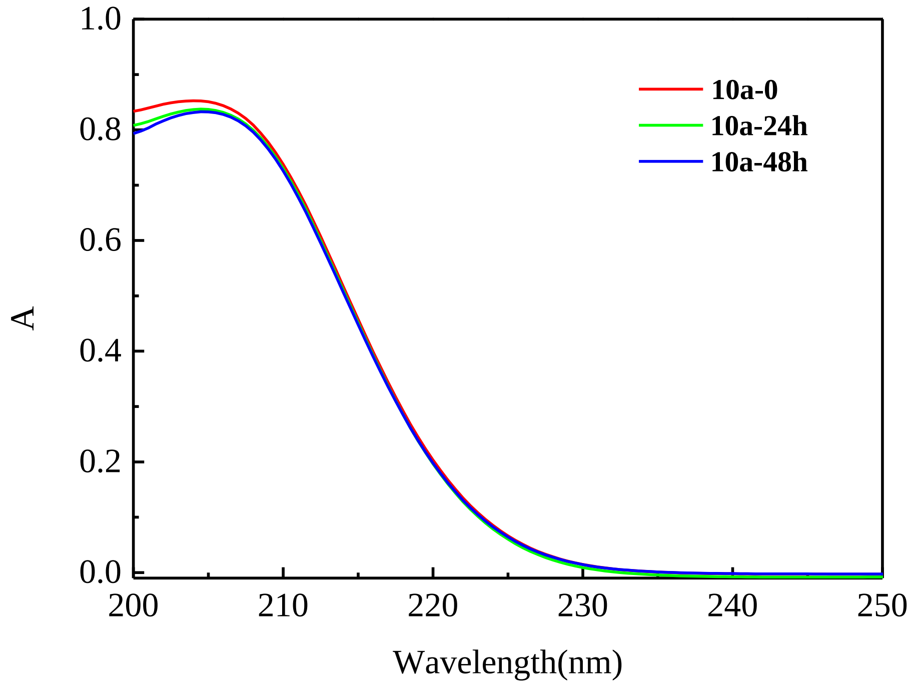

Figure S26. The UV-vis spectra of **10a** within 48 h in Tris-HCl buffer (pH 7.4).

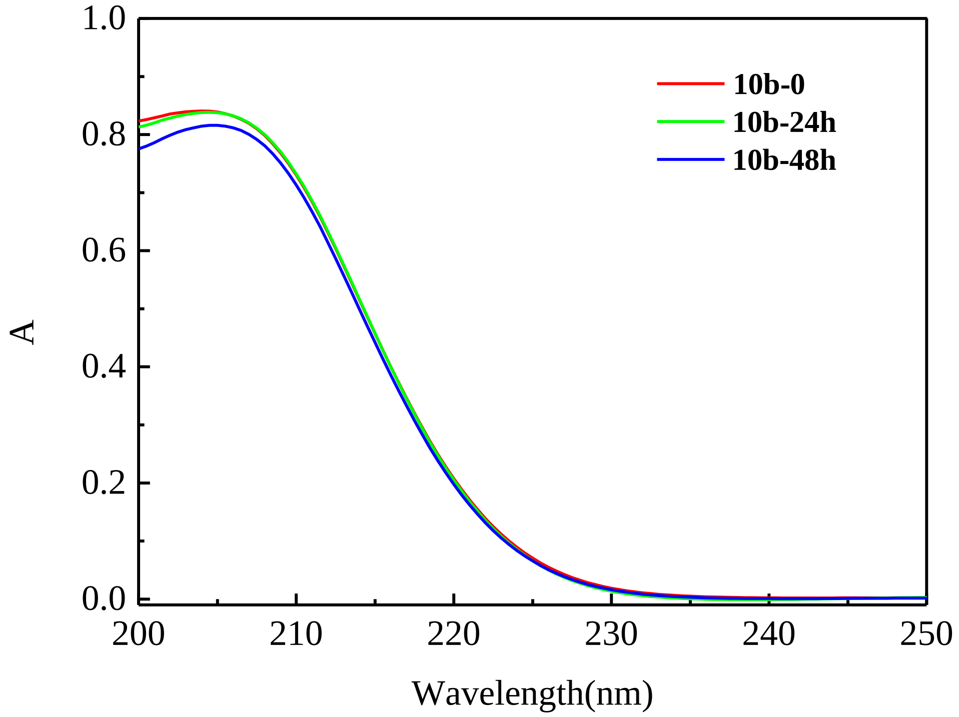

Figure S27. The UV-vis spectra of **10b** within 48 h in Tris-HCl buffer (pH 7.4).

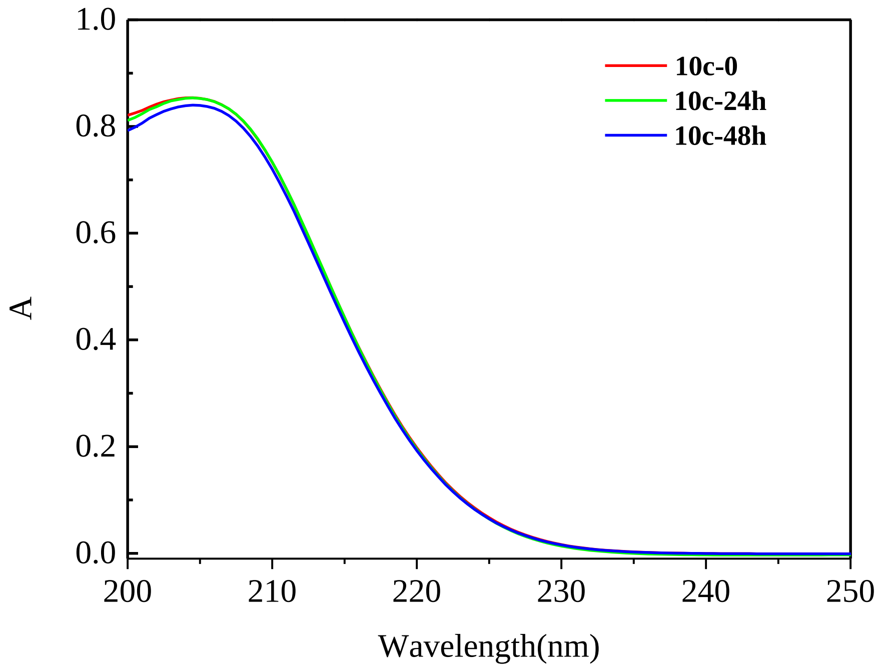

Figure S28. The UV-vis spectra of **10c** within 48 h in Tris-HCl buffer (pH 7.4).

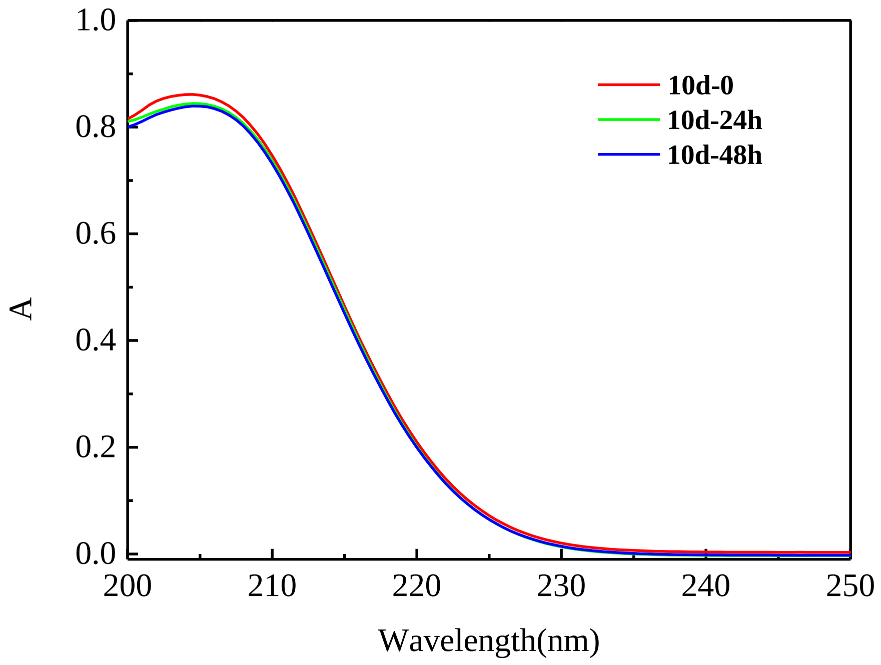

Figure S29. The UV-vis spectra of **10d** within 48 h in Tris-HCl buffer (pH 7.4).

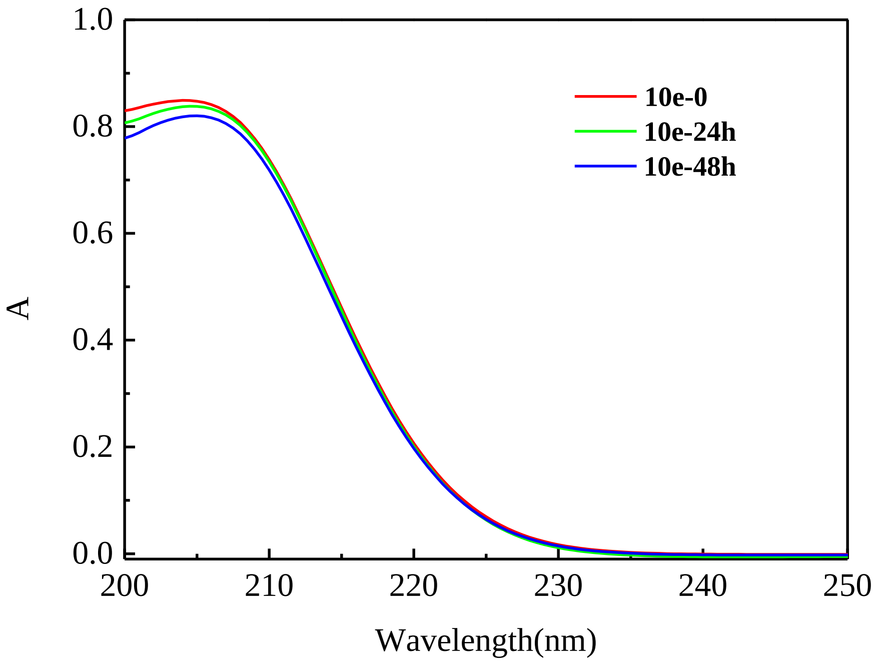

Figure S30. The UV-vis spectra of **10e** within 48 h in Tris-HCl buffer (pH 7.4).

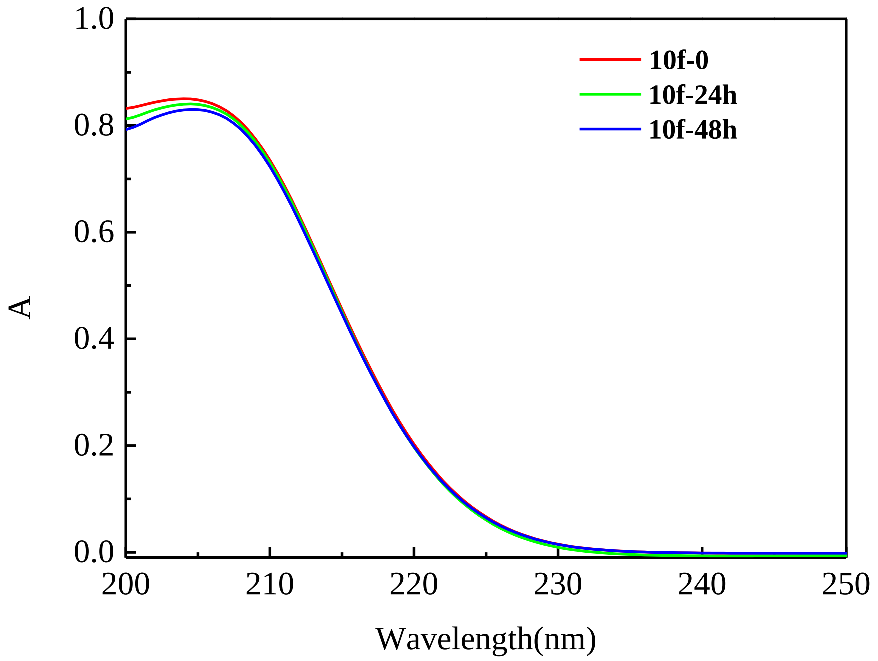

Figure S31. The UV-vis spectra of **10f** within 48 h in Tris-HCl buffer (pH 7.4).

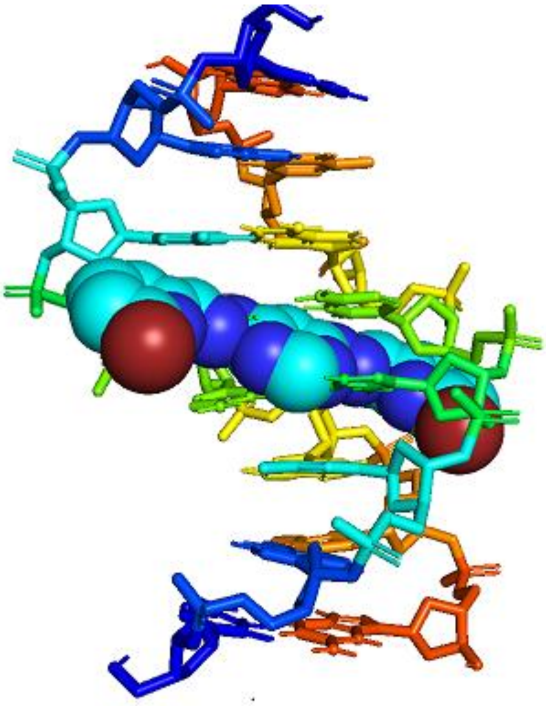

Figure S32. Visualizations of **10a** docking with DNA (PDB ID: 1D18)

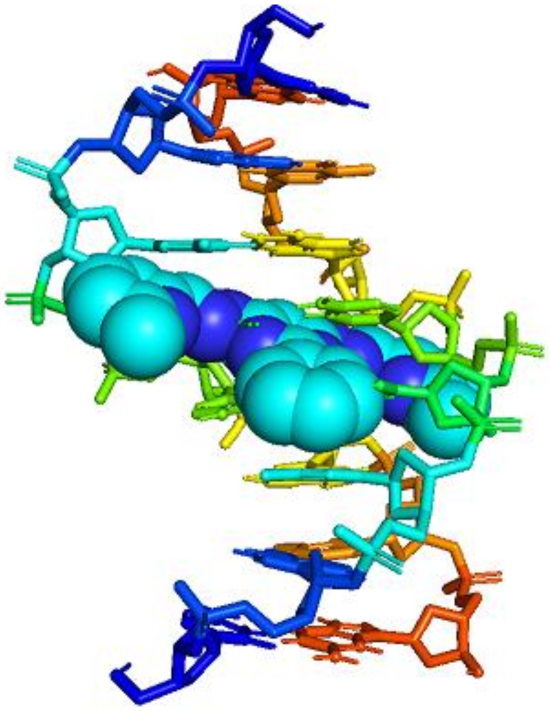

Figure S33. Visualizations of **10f** docking with DNA (PDB ID: 1D18)

Table S1. The ΔG_b^0 and K_b of **10a** and **10f** interacting with DNA (PDB ID: 1D18).

Compound	ΔG_b^o (kcal/ mol)	K_b (M^{-1})
10a	–8.0	7.75×10^5
10f	–8.1	9.18×10^5
5-FU	–5.0	4.81×10^3
